# Cingulum and Uncinate Fasciculus Microstructural Abnormalities in Parkinson’s Disease: A Systematic Review of Diffusion Tensor Imaging Studies

**DOI:** 10.3390/biology12030475

**Published:** 2023-03-20

**Authors:** Fatemeh Rashidi, Mohammad Hossein Khanmirzaei, Farbod Hosseinzadeh, Zahra Kolahchi, Niloofar Jafarimehrabady, Bardia Moghisseh, Mohammad Hadi Aarabi

**Affiliations:** 1 School of Medicine, Tehran University of Medical Science, Tehran 1417613151, Iran; 2 Department of Clinical-Surgical, Diagnostic and Pediatric Sciences, University of Pavia, 27100 Pavia, Italy; 3 School of Medicine, Arak University of Medical Science, Arak 3848176941, Iran; 4 Department of Neuroscience (DNS), Padova Neuroscience Center, University of Padova, 35128 Padua, Italy

**Keywords:** diffusion tensor imaging (DTI), Parkinson disease (PD), cingulum, uncinate fasciculus, non-motor symptoms, motor symptoms

## Abstract

**Simple Summary:**

This article reviews the use of diffusion tensor imaging (DTI) to evaluate changes in white matter microstructure within two specific fiber tracts in Parkinson’s disease patients. It also examines how these structural changes may be related to cognitive impairments seen in advanced PD patients and provides insight into developing more targeted treatments for different types of Parkinson’s disease.

**Abstract:**

Diffusion tensor imaging (DTI) is gaining traction in neuroscience research as a tool for evaluating neural fibers. The technique can be used to assess white matter (WM) microstructure in neurodegenerative disorders, including Parkinson disease (PD). There is evidence that the uncinate fasciculus and the cingulum bundle are involved in the pathogenesis of PD. These fasciculus and bundle alterations correlate with the symptoms and stages of PD. PRISMA 2022 was used to search PubMed and Scopus for relevant articles. Our search revealed 759 articles. Following screening of titles and abstracts, a full-text review, and implementing the inclusion criteria, 62 papers were selected for synthesis. According to the review of selected studies, WM integrity in the uncinate fasciculus and cingulum bundles can vary according to symptoms and stages of Parkinson disease. This article provides structural insight into the heterogeneous PD subtypes according to their cingulate bundle and uncinate fasciculus changes. It also examines if there is any correlation between these brain structures’ structural changes with cognitive impairment or depression scales like Geriatric Depression Scale-Short (GDS). The results showed significantly lower fractional anisotropy values in the cingulum bundle compared to healthy controls as well as significant correlations between FA and GDS scores for both left and right uncinate fasciculus regions suggesting that structural damage from disease progression may be linked to cognitive impairments seen in advanced PD patients. This review help in developing more targeted treatments for different types of Parkinson’s disease, as well as providing a better understanding of how cognitive impairments may be related to these structural changes. Additionally, using DTI scans can provide clinicians with valuable information about white matter tracts which is useful for diagnosing and monitoring disease progression over time.

## 1. Introduction

Parkinson’s disease (PD) is a prevalent neurodegenerative disorder that affects 1% of individuals over the age of 60 worldwide [1,2,3]. Symptoms of PD can be divided into two categories: motor and nonmotor. Motor symptoms, which are the most well known, include akinesia (reduced movement), bradykinesia (slowness of movement), tremor, rigidity, gait disturbance, and speech deficits [4]. In recent years, nonmotor symptoms of PD have received increased attention. These symptoms include autonomic dysfunction, sensory issues, cognitive impairment, sleep disturbances, apathy, depression, and anxiety [5,6]. From a pathophysiological perspective, it is believed that these symptoms result from the loss, degeneration, and impaired neurogenesis of dopaminergic neurons in specific regions of the brain, such as the basal ganglia, the substantia nigra, the hippocampus, the limbic system, and the white matter [7,8,9,10].

Studies utilizing diffusion tensor imaging (DTI) on animal models of Parkinson’s disease induced by toxins, resulting in the loss of dopaminergic neurons in the substantia nigra (SN), have yielded inconclusive results regarding changes in DTI measures [11,12].

**Figure 1:**
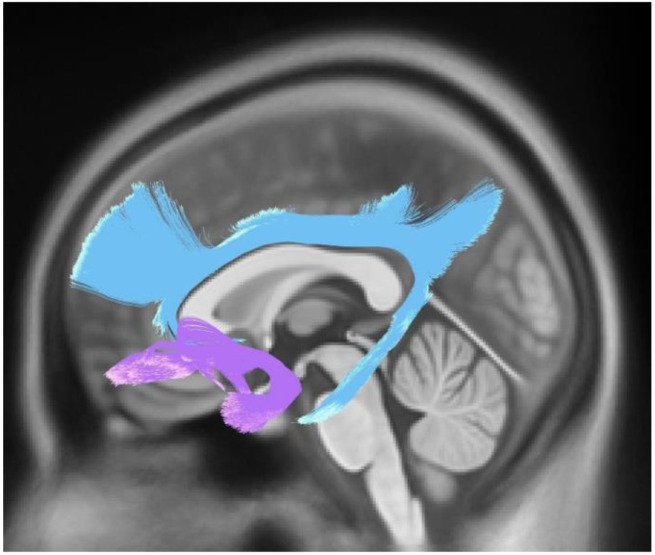
Tractography of cingulum (blue) and uncinate fasciculus (purple).

White matter (WM) degeneration is a commonly observed magnetic resonance imaging (MRI) finding in PD, and it is characterized by abnormal fiber connections in the brain. This degeneration can affect various regions of the brain, including the prefrontal limbic system. This degeneration might also lead to the cognitive impairment and changes in emotion regulation often seen in PD patients [13,14]. Abnormal white matter fibers have been suggested as a potential cause of depression in PD. The limbic system, a complex of brain structures that plays significant roles in memory, language, goal-directed behavior, affective behavior, and emotional behavior, is also affected in PD. The limbic system includes structures such as the cingulate gyrus, hippocampus, parahippocampal gyrus, amygdala, mammillary bodies, hypothalamus, and the nucleus accumbens [15,16]. The uncinate fasciculus (Figure 1), which connects the inferior frontal gyrus with the anterior temporal lobe regions, is traditionally a considered part of the limbic system [17]. The cingulum (Figure 1), a bundle of nerve fibers that runs from the orbital frontal cortex, along the corpus callosum’s dorsal surface, and down the temporal lobe, is also an integral part of the limbic system [18,19].

Recent MRI studies have revealed dysfunction of the cingulum in a variety of neurological and psychiatric disorders. Despite limited available data on anatomy and function of the cingulum, it is crucial to unravel hidden interactions of the highly complex limbic system [20]. DTI is a technique used to assess the microstructural composition of white matter by analyzing the movement of water molecules in the brain. It has been used to detect changes in the brain tissue caused by neurological diseases [21]. Intact WM contributes to high fractional anisotropy (FA), which results from high directionality of water diffusion along axon bundles and lower tissue density [22]. Mean diffusivity (MD) is also used to assess the diffusion of water [23]. Additionally, diffusion can be measured parallel or perpendicular to white matter fascicles, known as axial diffusivity (AD) and radial diffusivity (RD), respectively. Changes in AD values indicate axonal damage and fragmentation, while changes in RD values indicate changes in axonal density, axonal diameter, and myelination [23].

DTI has been widely used to investigate pathological changes in the WM of PD patients by probing the diffusivity of water molecules within the WM tracts [24].

In this systematic review, we aim to provide a comprehensive understanding of specific WM fiber tracts, particularly the cingulum bundle and uncinate fasciculus, in Parkinson’s disease patients, in terms of their associations with DTI profiles.

## 2. Search Strategy and Data Extraction

**Figure 2:**
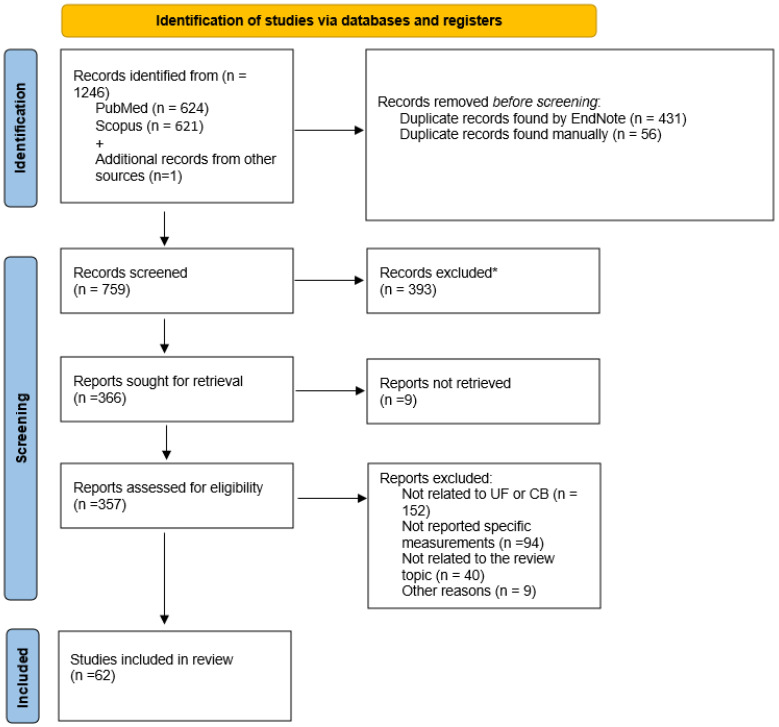
PRISMA flow diagram for DTI of the cingulum and uncinate fasciculus in PD. *, studies which had investigated diseases other than PD or had used imaging methods other than DTI were excluded.

We performed a systematic search of the published literature to identify the studies that investigated the involvement of uncinate fasciculus and cingulum associated with PD pathology and symptomatology using DTI. We used the broad search terms: “Diffusion Tensor imaging” [All Fields] OR “Diffusion tensor MRI” “[All Fields] OR” “ Diffusion MRI” “[All Fields] OR” “ DTI ”[All Fields]) AND (“Parkinson’s disease “[All Fields] OR ” Parkinson disease “[All Fields] OR ” PD “[All Fields])”. We searched electronic databases including Scopus and PubMed from 2015 to November 2022, This was complemented by manual searching of the related papers through the list of references. To avoid duplication, the results were imported to Covidence software and articles were separately screened by two of the authors (Z.K. and M.H.K.). In case of disagreement, a third person (F.H.) interfered to decide whether to include or exclude articles. Among the search results, abstracts were screened for relevance. Studies which had investigated diseases other than PD or had used imaging methods other than DTI were excluded. Full papers were obtained for studies published in English that performed DTI in PD patients, and further assessed if they had investigated the cingulate bundle and uncinate fasciculus in PD patients to be included in this systematic review. Figure 2 illustrates the process of study selection according to the PRISMA guidelines.

## 3. Result

In our literature review, we identified 61 studies that investigated the changes in the uncinate fasciculus and cingulum in PD and used the PRISMA guidelines. One additional study was found during the writing process [25].

The studies were conducted globally, with inclusion ranging from 2015 until December 2022, However, several studies examining white matter changes in PD patients were excluded as they were either animal studies. Additionally, any studies that focused on structures other than the uncinate fasciculus and cingulum were also excluded. A summary of the study demographics is presented in Table 1.

All studies except ten [36,40,53,60,63,73,82,84,85,86] included healthy controls.

The sample size of the PD patients varied widely, with a pilot study having only seven female participants [34] and the largest sample size being 205 from the prospective and longitudinal Swedish BioFINDER study [31].

The studies included both male and female participants, with most studies having a predominantly male population. One study [34] only included females, and two studies did not mention the gender distribution [43,45].

The disease duration of Parkinson’s disease ranged from 1 ± 1.3 years in one study [60] to 14.3 ± 7.75 years in another study [35].

## 4. Cingulum

### 4.1. PD

A summary of the studies is presented in Table 2. PD is a progressive, degenerative disorder that affects multiple systems in the body. It is characterized by the accumulation of α-synuclein protein in various brain regions, leading to both motor and non-motor symptoms [31].

A previous study utilized DTI to demonstrate degeneration of the nigrostriatal pathway in PD patients. Results showed differences in FA and MD, highlighting the value of DTI in the diagnosis of PD [31].

A study conducted on animals examined the effectiveness of diffusion kurtosis imaging (DKI)—an extension of DTI—in detecting changes caused by the accumulation of α-synuclein (α-syn) in the white matter (specifically the cingulum) of α-syn over-expressing transgenic mice (TNWT-61). The findings suggest that DKI could serve as a highly sensitive method for identifying changes in brain tissue induced by α-synuclein accumulation, which may indicate the progression of Parkinson’s disease [31].

The cingulum, being a vulnerable area in the brain, has drawn attention in neurodegenerative research. Research has suggested that evaluating the cingulum fibers through DTI could enhance early diagnosis of neurodegenerative diseases.

Decreased connectivity in the cingulum tract has been found to be negatively correlated with neutrophil-to-lymphocyte ratio (NLR) in the early stage of PD progression [31].

NLR is a non-invasive marker of peripheral neuroinflammation and increased NLR is associated with poor cellular immunity.

According to the results of the study, degeneration of central white matter tracts in the brain occurs early in Parkinson’s disease and is primarily located in the cingulum. This degeneration may contribute to early cognitive dysfunction. Changes in the DTI measures, including increased MD [31,39,64,68] and decreased FA [50,59,65] have been detected in PD patients, with a higher number of group differences being found as the mean diffusivity increases.

Studies have suggested that MD may be more sensitive in detecting subtle white matter changes in early PD than FA, as has been found in other studies in early Alzheimer patients [87].

The pattern of decreased FA and increased MD and RD is indicative of neurodegeneration [48], which has been found in individual PD patients in the present study [33].

Three studies [34,52,70] suggest that the cingulum (where the FA of PD patients is greater than the FA of controls) is modulated by PD through a compensatory mechanism. The FA measures obtained from these brain regions may potentially be used to detect brain signal changes in an early stage of PD, possibly even before the clinical manifestation of motor symptoms [34].

The exact cause of the changes observed in the DTI of PD patients remains unknown, but it is believed to be due to variations in the diffusion ellipsoid dimensions caused by the neurodegenerative process. While FA is commonly used as a measure of white matter integrity, this interpretation should be approached with caution as it is influenced by various factors such as myelination, axon packing, membrane permeability, internal axonal structure, and tissue water content [88].

The findings of some studies have shown decreased FA and increased RD [45] in the cingulum of PD patients, while others have found increased FA and decreased AD in the same region [42]. It is hypothesized that extensive damage to white matter fibers occurs in the early stages of PD, potentially due to the aggregation of synapsins and Lewy bodies in vulnerable brain regions, resulting in atrophy, neuron loss, and demyelination of nerve fibers [89].

### 4.2. Motor Symptoms

In comparison to healthy controls (HC), most studies found degeneration in PD patients with motor symptoms, as indicated by decreased FA [37,79,83], increased MD [74] and a combination of decreased FA and increased MD [46]. These results suggest that FA, MD, and other DTI measures could serve as quantitative biomarkers of motor symptom severity in PD. Lower FA is typically associated with decreased WM connectivity and is considered an indication of WM microstructural abnormalities. However, it is unclear why these changes occur. Motor symptoms usually appear late in PD patients, which suggests that decreases in FA occur later in the disease than increases in MD. Some studies have found increased FA in PD patients [52,60].

The cingulum, an association fiber that connects anterior and posterior cortical regions, showed increased FA or decreased MD/RD in PD patients and was associated with better olfaction and lower motor severity [76,90].

These findings suggest that increased connectivity in these WM structures could serve as a compensatory mechanism to facilitate efficient information transfer between different regions of the brain.

### 4.3. Non-Motor Symptoms

It has been found in most studies that individuals with PD who have non-motor symptoms such as dementia, depression, cognitive impairment, psychosis, or apathy have significantly decreased FA values compared to HC [29,36,40,56,80,86].

The pattern of decreased FA and increased mean diffusivity (MD) has also been shown in several studies [33,47,62].

In some studies, compensation was also defined by increased FA or decreased MD [43,55,84].

Studies have consistently shown that an increase in MD (AD and RD) is associated with cell atrophy and demyelination, which may indicate extensive degeneration in advanced PD patients who present with dominant non-motor symptoms. This loss of structural organization is believed to be linked to neurodegeneration.

Depression and dementia frequently occur in PD patients, often appearing late in the disease at stage 3 or 4 of the Hoen and Yahr staging for motor involvement. They can also be present early on in the honeymoon period of PD, and have been shown to correlate with the severity of motor involvement [37]. Abnormal functioning in depression and dementia in PD patients may be due to degeneration of the microstructure in the white matter located in frontal-limbic regions. This has been observed in previous studies, and one hypothesis is that abnormalities in the frontal limbic system cause depression in PD patients [27].

Disruption of the structural integrity of white matter in the cingulum tract can be recognized as a marker to predict early PD, regardless of white matter alteration related to REM sleep disorder [83,91], depression [91,92], or olfaction dysfunction [93], which are thought to be early nonmotor symptoms of PD.

Thus, changes in the cingulum microstructure could be used to detect early stages of PD and help distinguish between PD patients without dementia and depression or those in preclinical stages.

### 4.4. Correlation

DTI values in the cingulum have been shown to be significantly associated with cognitive function in PD patients. This was demonstrated by a correlation between DTI values and scores from the Mini-Mental State Examination (MMSE) and the Frontal Assessment Battery (FAB). The study results indicate that the more extensive the diffusivity abnormalities in the cingulum, the worse the cognitive performance [66].

Spearman rank order correlation analyses found significant correlations between changes in FA values in the cingulum and sociodemographically corrected Consortium to Establish a Registry for Alzheimer’s Disease (CERAD) total scores in PD patients [29].

Additionally, lower scores on the Parkinson’s Disease-Cognitive Rating Scale (PD-CRS) were associated with decreases in FA values in the cingulum [63]. A novel finding from one study showed a linear association between AD and the PD-CRS score in major WM tracts, without concurrent RD alterations. This suggests that extensive and progressive axonal degeneration, without evident demyelination, may be involved in cognitive impairment in PD [94,95].

The cingulum bundle has a correlation with the short Geriatric Depression Scale (GDS) [42].

In one study, the FA values in the left cingulum of PD patients with depression were negatively correlated with the Hamilton Depression Rating Scale (HDRS) scores, but no correlation was found with other disease characteristics such as age, duration, Unified Parkinson Disease Rating Scale III (UPDRS III), H&Y scale, and MMSE [36]. Another study found that the FA values negatively correlated with the UPDRS-III scores across all PD patients in the cingulum [34]. Positive correlation with disease duration and RD in the left cingulum was revealed by using Tensor-based registration (DTI-TK) and negative correlation by TBSS. The study suggested a preference for the DTI-TK based registration technique before statistical analysis [61].

On the other hand, some studies found no significant correlation between FA in the left cingulum and clinical measures [50,73].

A significant association was also found between FA of the ROI in the left cingulum and appendicular skeletal muscle mass index (ASMI). Low FA values in the left cingulum were identified as the strongest predictor of sarcopenia in PD patients [53]. Positive correlations of FA and non-motor symptoms such as depressive symptoms were also found in the left cingulum in some studies [85].

Interestingly, it has been found that PD risk can be affected by cardiovascular risk factors including serum cholesterol and apo-lipoprotein levels [96].

In the early stages of PD, apo-lipoprotein A1 might predict the microstructural changes of certain white matter tracts like the cingulum [97].

The relationships between clinical presentations, MD, AD, RD, and serum nuclear DNA levels have also been demonstrated. Results suggest that poor cardiovascular autonomic status in PD patients not only directly affects the white matter microstructure but also increases the serum nuclear DNA level, further impacting the white matter microstructure [79].

Impairment of the ipsilateral posterior cingulum in PD may reflect the loss of dopaminergic inputs from the midbrain, as indicated by the statistically significant association between Activities of Daily Living (ADL) and maximum MD/RD [81].

A correlation between verbal memory and FA in the right posterior cingulum tract (PCT) was found, with greater FA in the right PCT being associated with better performance in verbal recognition memory, a core process in subsequent recognition memory [56].

The Lille Apathy Rating Scale (LARS) scores of the apathetic PD group were negatively correlated with the FA values in the left cingulum [86].

The AD and RD values were positively associated with the UPDRS, UPDRS-III, and NMSS scores in the cingulum [45].

A negative association between RD and a positive association between FA values and the Scales for Outcomes in Parkinson’s disease—Cognition (SCOPA-COG) scores were found in the cingulum [45].

One study found correlations between the MD parameter and declining processing speed and discrepancies in the cingulum tract [31].

## 5. Uncinate Fasciculus

### 5.1. PD

A summary of the included studies is presented in Table 3. The uncinate fasciculus interacts with the orbitofrontal cortex, assigning value to stored representations through interactions with temporal lobe-based information related to reward and punishment [98].

The most common cause of PD in autosomal recessive families is mutations in the parkin gene (PRKN) [99].

Tract-based spatial statistics (TBSS) using permutation analysis of linear models (PALM) has revealed elevated radial diffusivity (RD) in patients with parkin dysfunction (PRKN) compared to HCs. This finding is considered to be one of the most prominent pathological manifestations of parkin dysfunction, as it has been demonstrated by the elevated RD in multiple tests [57]. Another study [72] also confirms these results and suggests that PRKN patients with widespread increases in RD are more susceptible to widespread demyelination.

Different methods, including convolutional neural network (CNN) based methods, have shown that patients with PD exhibit increased mean diffusivity (MD) values [35,38,49,51,64]. However, some studies have reported decreased fractional anisotropy (FA) values [44,48,59,67]. MD appears to be more sensitive at detecting subtle changes in white matter in the early stages of PD compared to FA. It is due to damages to axons and neurons, as well as loss of myelin integrity in PD, that might result in decreased restriction of water molecule displacement, leading to higher MD values in PD patients [100].

A distinct pattern of neurodegeneration, characterized by low FA, high MD, low AD, and high RD, has been identified in PD patients [48]. This pattern was observed in a study by Andica et al. using TBSS [32] suggesting that PD patients are more susceptible to degeneration of the uncinate fasciculus (UF).

Christina Andica et al. conducted an analysis of white matter (WM) and gray matter in PD patients using TBSS. They found that PD patients with neurocognitive and psychiatric disorders (PD-wNCP) and PD patients without these disorders (PD-woNCP), compared to healthy controls (HCs), exhibited lower fractional anisotropy (FA), higher mean diffusivity (MD), higher radial diffusivity (RD), and higher axial diffusivity (AD), which has been described as neurodegeneration [33]. This pattern has previously been defined as neurodemyelination [48].

### 5.2. Motor Symptoms

The exact role of the UF in the development of motor symptoms in Parkinson’s disease is still not clear. Only a few studies have investigated the effect of the uncinate fasciculus on motor symptoms, with inconsistent results [28,66,71].

### 5.3. Non-Motor Symptoms

Parkinson’s disease, along with other neurodegenerative diseases such as Alzheimer’s disease, frontotemporal dementia, and apathy, is characterized by non-motor symptoms that are associated with white matter (WM) pathways, including the UF and cingulum [101].

Changes in diffusion tensor imaging (DTI) measures have been observed in the UF in PD patients with cognitive impairment, depression, and apathy. This suggests that non-motor symptoms in PD are related to the impairment of long white matter nerve fibers. It is known that multiple neurotransmitter pathways, including noradrenergic and cholinergic pathways, that project to the frontal lobe are impaired in PD patients with non-motor symptoms and other non-cognitive problems [102,103,104,105,106].

Studies have reported that patients with non-motor symptoms such as depression, dementia, and cognitive impairment exhibit more degeneration, as indicated by decreased FA [36,63,80], increased MD [26,73], and increased mean diffusivity and AD [27].

A significant reduction in white matter connectivity in UF has been found in PD patients with depressive symptoms compared to non-depressed patients. The pathophysiology of depression has been extensively studied in relation to UF, with reduced FA serving as a marker for tract microstructural alteration in individuals with major depressive disorder (MDD) [107]. Although Delaparte et al. [108] could not identify any significant differences between anxious and non-anxious depression, anxiety, which is frequently associated with depression, has been demonstrated to be connected to disrupted UF [108,109].

Previous research [23,110,111] has defined degeneration in the UF as low FA and high MD in patients with impulsive–compulsive behaviors and PD with neurocognitive and psychiatric symptoms [33,54].

In addition, findings of increased MD confirm neurodegeneration observed in prior studies. One study found that PD patients with impulse control behaviors (PD-ICB) had higher MD compared to HCs in the UF [35].

### 5.4. Correlation

Alterations in DTI parameters in PRKN patients were closely linked to both disease duration and serum levels of 9-hydroxystearate, a marker of oxidative stress. The microstructural changes in white matter seen in PRKN patients may therefore be a result of disease duration and oxidative stress. The study by Koinuma et al. [57] showed that the AD values in the UF were negatively correlated with the serum levels of 9-hydroxystearate, while MD and RD values were positively correlated with these levels.

A study found a correlation between the uncinate fasciculus and access to lexical semantic information stored in the temporal lobe, primarily in the left hemisphere. The results suggest that both the right and left UF support word production when selecting among competing alternatives is required [38].

Another study found a significant positive correlation between brain activation in the left IOFC during the verbal learning memory fMRI task and the FA of the right UF. This suggests that the greater the integrity of the UF in PD patients, the greater the functional brain activation in the left IOFC while performing the learning task. The study also revealed a significant correlation between brain activation in the left inferior orbitofrontal cortex (IOFC) during the verbal recognition functional magnetic resonance imaging (fMRI) task and verbal memory impairment, suggesting that the deficit in verbal memory performance during the fMRI paradigm could be influenced by lower brain activation in orbitofrontal cortices during the recognition memory fMRI task [56].

In one study, it was found that there was no relationship between UPDRS and motor scores with the FA of each white matter fasciculus [59].

However, in another study, the FA values were negatively correlated with the UPDRS-III scores across PD patients in the UF [34].

A decrease in total PD-CRS score was associated with decreased FA values in the UF [63]. However, no significant correlation was found between BDI scores and FA values [73].

Additionally, a significant correlation has been observed between the DTI values in the right UF and the Hamilton Depression Scale (HAM-D) scores [66].

In addition, one study found a correlation between MD parameter and MoCa scores in the UF [26].

Based on several studies, the correlation between changes in white matter tracts and cognitive impairment does not seem to be influenced by region, cell type, or gender. Additionally, some studies have reported that voxel-wise correlation analysis for fractional anisotropy (FA) values did not reveal any variations based on either cell type or gender [29].

Further investigation comparing patients with and without Parkinson’s disease (PD) found no significant differences in terms of age, gender, or level of education [39]. In addition, the results of multiple linear regression analyses indicated that in people with Parkinson’s disease, white matter (WM) integrity and being male were significantly associated with muscle mass [53].

Studies conducted on various diseases, including Alzheimer’s disease (AD), have demonstrated that patients may experience changes in brain structure even before displaying symptoms of cognitive impairment [112]. Likewise, the studies we have included suggest that diffusion tensor imaging (DTI) may be useful in detecting microstructural changes in Parkinson’s disease before clinical symptoms become apparent [28,29,32,38,39,46,53,58,81,112].

Several studies suggest that in the early stages of Parkinson’s disease, neural reorganization may occur as a compensatory mechanism to combat the pathology. This phenomenon could potentially explain why some individuals with Parkinson’s disease do not experience cognitive impairments [76].

DTI may not be able to detect early changes in Parkinson’s disease, but it can potentially serve as a surrogate marker by differentiating between early and late stages of the disease [45].

To confirm these findings and investigate potential links between preclinical brain changes and later development of cognitive impairment symptoms in Parkinson’s disease patients, a longitudinal study is necessary.

Earlier research has suggested that diffusion tensor imaging (DTI) could serve as a diagnostic tool to differentiate Parkinson’s disease patients from healthy individuals. By analyzing white matter fiber connections and measuring specific biomarkers, DTI may be capable of providing clinical presentations and assessing the severity of Parkinson’s disease [113,114].

As we mentioned before, FA, MD, and other DTI measures could serve as quantitative biomarkers of motor and non-motor symptoms in PD patients. Despite abundant published studies of DTI markers in PD, DTI is not currently widely utilized in clinically standard MRI scanning [115].

Due to limited scanning time, conducting DTI in clinical setting may result in problems such as noise, fiber crossings, low resolution, distortion, and artifacts. Thus, the decreased quality of DTI images makes it hard to obtain precise quantitative measurements [116,117].

The quality of DTI analysis will increase with the use of advanced diffusion techniques, including high-resolution, high-field MRI, enhanced distortion corrections, and fiber crossing solutions [118,119].

However, it is crucial to create clinically useful parameters based on these cutting-edge methods. Furthermore, scanning parameters including MRI field strength, number of encoding directions, and maximum b values have a significant impact on DTI variables. DTI measurements from various MRI facilities need to be harmonized, and consistent cutoff values for these DTI parameters need to be created in order to eventually improve the individual definition and treatments of PD.

## 6. Conclusions

Our review provided microstructural insight into the heterogeneous PD subtypes according to their distinct clinically relevant connectivity features.

Cingulum: In this study, we found that individual PD patients had increased MD, possibly defined by degeneration in the early stages. When PD patients experience motor symptoms, FA decreases and/or MD increases, which may result in more degeneration at a later stage of the disease. PD patients with non-motor symptoms showed significant decreases in FA more towards the end of the disease, indicating that extensive degeneration occurred in their non-motor symptoms.

UF: There is a high probability of widespread demyelination and degeneration in UF for PD.

Non-motor symptoms appear lately with extensive degeneration. Cingulum compensation occurred for both motor and non-motor symptoms similarly.

## Figures and Tables

**Table 1:** An overview of the demographic of literature regarding studies with significant microstructural changes of Cingulum and Uncinate Fasciculus in association with PD symptoms.

	Demographic Features			
Study	Groups Studied	Number of Participants (Male)	Mean Age ± SD (Years) or Range	Disease Duration ± SD (Years) or Range
**Boyu Chen et al., 2015** [26]	HC PD-Cu PDD	21 (11) 19 (10) 11 (6)	61.10 ± 8.336 59.47 ± 8.771 64.09 ± 11.353	– 3.21 ± 1.960 3.64 ± 2.693
**Zonghong Li et al., 2020** [27]	HC non-depressed PD depressed PD	91 (40) 43 (24) 30 (13)	57.67 ± 5.27 58.09 ± 6.52 59.23 ± 7.10	– 6.28 ± 3.38 5.46 ± 4.25
**Florian Holtbernd et al., 2019** [28]	PD HC RBD	29 (21) 56 (42) 30 (29)	63.5 ± 8.3 62.9 ± 11.0 66.8 ± 9.1	80.9 ± 85.7 (months) _ 121.0 ± 162.4
**Martin Gorges et al., 2019** [29]	HC PD	72 (39) 134 (98)	65 ± 7 67 ± 8	9 ± 4 9 ± 5
**Junyan Sun et al., 2021** [30]	PD HC	68 (36) 77 (30)	58.94 (8.969) 59.58 (8.537)	_ 4 (0.5–20)
**Mattis Jalakas et al., 2019** [31]	HC PD PDD	51 (0.56) (1 = female) 175 (0.37) 30 (0.3)	65 (8.5) 65 (10) 72.5 (6.6)	NA 5.1 (4.9) 14 (6.8)
**Christina Andica et al., 2019** [32]	HC PD	20 (12) 20 (11)	67.15 1.18 65.05 10.9	– 6.95 3.93
**Christina Andica et al., 2020** [33]	HC PD-woNCP PD-wNCP	25 (10) 19 (6) 20 (12)	67.88 ± 2.11 67.21 ± 8.16 70.15 ± 4.03	– 9.84 ± 6.21 9.95 ± 7.69
**Nan-Kuei Chen et al., 2018** [34]	PD HC	7 (0) 15 (0)	64.03–10.30 58.03–9.28	62.9–43.6 (months)
**Haruka Takeshige-Amano et al., 2022** [35]	HC PD-ICB PD-nICB	20 (9) 19 (10) 18 (5)	66.75 1.07 67.11 7.00 66.28 5.03	– 14.3 7.75 10.2 4.82
**Jia-Yong Wu et al.**, **2017** [36]	PDD PDnD	31 (18) 37 (23)	58.8 ± 8.67 59.1 ± 11.4	3.23 ± 3.04 2.40 ± 2.53
**Jingwen Chen et al., 2022** [37]	HC LAR LTD RAR RTD	50 (28) 38 (17) 32 (16) 43 (25) 48 (34)	59.2 ± 6.3 59.4 ± 10.3 60.4 ± 8.9 57.9 ± 10.6 59.3 ± 9.7	– 4.5 ± 4.5 3.9 ± 3.1 3.4 ± 3.2 4.5 ± 4.2
**Sonia Di Tella et al., 2020** [38]	HC PD	17 (9) 19 (10)	64.08 (12.71) 67.75 (13.25)	NA 29.00 (38.00) (months)
**Gordon W. Duncan et al., 2015** [39]	HC PD	50 (29) 125 (85)	65.8 ± 8.0 66.0 ± 10.5	6.15 (4.66)
**Abhishek Lenka et al., 2020** [40]	PD-NP PD-P	48 (41) 42 (35)	57.9 ± 7.0 58.5 ± 7.8	5.7 ± 2.4 6.5 ± 3.2
**Rémi Patriat et al., 2022** [41]	HC PD−RSWA PD + RSWA	21 (10) 20 (14) 18 (9)	61.3 + 8.0 63.0 + 8.6 65.8 + 4.8	– 2.1 + 1.5 2.8 + 2.2
**Yuanjing Feng et al., 2020** [42]	SWEDD PD HC	42 (27) 50 (35) 50 (35)	60.7 ± 9:4 60.3 ± 8:9 60.6 ± 10:3	6.9 ± 8:0 6.7 ± 7:3 NA
**Markus Nilsson et al., 2015** [43]	PDD HC	11 (NA) 27 (NA)	74 ± 7 70 ± 4	NA
**Lubin Gou et al., 2018** [44]	d-PD nd-PD HC	28 (17) 56 (36) 37 (21)	61.43 ± 10.06 63.97 ± 8.31 60.35 ± 11.70	NA
**Rachel P. Guimarães et al., 2018** [45]	All PD MPD MoPD SPD HC	97 24 60 13 137 (83)	60.93 ± 9.8 61.66 ± 9.6 60.30 ± 10.69 61.96 ± 8.4 58 ± 9.39	7.8 ± 6.43 4.15 ± 4.08 8.74 ± 6.62 12.14 ± 5.31 –
**Chaoyang Jin et al., 2021** [46]	HC PD FOG + PD FOG–	24 (15) 24 (11) 37 (18)	62.5 ± 3.8 65.5 ± 6.1 64.1 ± 8.2	– 6.00 ± 5.25 3.01 ± 3.21
**Yunjun Yang et al., 2022** [47]	Dpd ndPD HC	37 (19) 35 (21) 25 (9)	60.73 ± 11.22 62.40 ± 11.10 57.08 ± 7.93	5.01 ± 3.01 3.57 ± 3.65 –
**Xiaojun Guan et al., 2018** [48]	HC PD	46 (21) 65 (32)	57.8 ± 9.4 55.5 ± 9.5	– 4.7 ± 3.9
**Elisa Canu et al., 2015** [49]	PD-FoG HC	23 35	66.9± 8.0 67.7± 7.6	≥5 years
**Gong-Jun Ji et al., 2019** [50]	PD HC	57 (29) 52 (20)	59.5 1.21 60.6 1.22	4.6 0.61 –
**Tao Guo et al., 2020** [51]	S-depression PD S-motor PD mild PD HC	53 (29) 37 (26) 44 (24) 77 (33)	60.89 ± 8.68 63.34 ± 10.05 59.38 ± 8.30 60.22 ± 7.40	3.86 ± 4.14 4.86 ± 3.03 3.77 ± 3.58 -
**Yiming Xiao et al., 2021** [52]	Left-dominant PD Right-dominant PD PD HC	62 (34) 79 (56) 141 (90) 62 (22)	59.8 ± 8.8 63.2 ± 8.8 61.7 ± 8.9 61.4 ± 9.8	NA
**Chih-Ying Lee et al., 2019** [53]	NC PDNSa PDSa	19 (10) 31 (9) 21 (12)	60.3 7.6 60.3 9.8 63.7 11.6	— 2.4 2.4 1.9 2.0
**F Imperiale et al., 2017** [54]	PD-ICB PD no-ICB HC	35 (30) 50 (36) 50 (35)	62.0 ± 10.4 61.5 ± 8.9 59.0 ± 12.4	9.5 ± 5.2 9.0 ± 6.1 NA
**Hye Bin Yoo et al., 2015** [55]	PD-ICD PD-nonICD HC	10 (7) 9 (6) 18 (10)	54.5 ± 6.2 59.6 ± 8.6 54.4 ± 6.5	10.2 ± 7.3 10.6 ± 3.9
**Olaia Lucas-Jim’enez et al., 2015** [56]	PD HC	37 (22) 15 (11)	67.97 (6.17) 65.07 (7.01)	6.96 (5.61) –
**Takahiro Koinumaa 2021** [57]	HC PARK2	15 (9) 9 (4)	55.2 (± 20.7) 58.3 (±14.1)	_ 27.5 (±11.9) months
**Charalampos Georgiopoulos et al., 2017** [58]	PD HC	22 (12) 13	68 (95% CI 67 and 70) 68 (95% CI 65 and 70)	7 (95% CI 5 and 9)
**Xiang-Rong Li et al., 2018** [59]	PD HC	31 22	60.5 ± 9.3 59.7 ± 8.6	NA
**Kazufumi Kikuchi et al., 2017** [60]	PD-MIBGH PD-MIBGL	12 12	66.8 ± 4.9 67.4 ± 6.1	1 ± 1.3 2 ± 1.9
**Virendra R. Mishra et al., 2019** [61]	HC PD	44 (29) 81 (52)	61 ± 10.79 61.35 ± 9.93	NA 11.46 ± 13.85
**Thais Minett et al., 2018** [62]	PD-NC PD-MCI HC	93 27 48	64.3 ± 10.8 70.5 ± 8.1 66.0 ± 7.9	6.4 ± 0.5 5.6 ± 0.7 -
**Maria Chondrogiorgi et al., 2019** [63]	PD-CTRL PDD	40 (31) 21 (16)	68.4 (6) 70.9 (5.7)	5.7 (4.8) 7.9 (6.8)
**Yuko Koshimori et al., 2015** [64]	HC PD	26 (13) 15 (4)	70.5 ± 5.6 67.13 ± 5.1	6.7 (4.2) –
**Ming-fang Jiang et al., 2015** [65]	PD HC	31 (16) 34 (18)	69.4 ± 8.0 69.3 ± 8.0	< 3 years in 15 cases, 3–5 years in 9 cases, and 5–10 years in 7 cases -
**Sara Pietracupa et al., 2017** [66]	PD-FOG PD-NFOG HC	21 (16) 16 (13) 19	66.3 ± 10.72 69,7 ± 11.1 66.74 ± 7.68	11 ± 6.3 9.5 ± 6.2 -
**A. Inguanzo et al., 2020** [67]	HC PD1 PD2 PD3	33 (18) 15 (13) 21 (14) 26 (19)	66 (15) 75 (14) 68 (9) 58.5 (11)	NA 7 (7.5) 9 (9) 7 (5.5)
**Laura Pelizzari et al., 2019** [68]	LPD RPD HC	9 (4) 12 (7) 17 (9)	68.3 (57.1–73.3) 70.2 (61–73.8) 64.1 (57.3–68.3)	4 (1.5–6) 2 (1–3.5) –
**Tracy R. Melzer et al., 2015** [69]	HC PD	23 (16) 23 (17)	70.6 ± 6.8 69.5 ± 6.4	_ 5.6 ± 4.3
**Yulia Surova 2016** [70]	HC PD	44 (19) 105 (44)	66 ± 8 66 ± 11	_ 5 ± 4
**Takashi Ogawa 2021** [71]	HCs PD-nLID PD-LID	23 (9) 26 (11) 25 (10)	67.0 ± 1.2 67.2 ± 4.7 66.8 ± 7.4	_ 7.3 ± 3.9 13.8 ± 7.1
**Jinqiu Yu et al., 2022** [72]	HC PD G/G G/A	28 (12) 26 (14) 27 (14) 12 (12)	62.3 ± 6 65.5 ± 6.8 63.9 ± 6.6 63.8 ± 6.6	NA
**Qin Shen et al., 2021** [73]	ndPD mdPD sdPD	30 (15) 22 (10) 15 (6)	56.9 ± 10.6 56.4 ± 8.0 57.8 ± 6.7	2.2 ± 1.2 2.3 ± 1.3 2.5 ± 1.5
**Morinobu Seki et al., 2019** [74]	PSP MSA-P PD HC	18 (14) 16 (8) 16 (9) 21 (8)	67.1 ± 6.5 63.9 ± 7.1 65.2 ± 5.3 62.3 ± 6.8	2.3 ± 1.5 1.9 ± 1.6 3.2 ± 2.0 _
**Min Wang et al., 2016** [75]	PD-FOG PD-nFOG HC	14 (9) 16 (10) 16 (8)	72.36 ± 6.15 68.88 ± 6.00 68.56 ± 2.56	3.29 ± 1.65 3.70 ± 2.94 _
**Ming-Ching Wen et al., 2018** [76]	HC TD PIGD	61 (41) 52 (32) 13 (10)	60.19 ± 10.80 60.46 ± 9.57 66.66 ± 10.17	7.52 ± 8.00 6.54 ± 6.78 -
**Jingqiang Wang et al., 2020** [77]	PD HC	30 (19) 28 (17)	59.3 ± 9.0 59.9 ± 9.7	NA
**Yang Zhang et al., 2017** [78]	Apathy Non-apathy	18 (17) 21 (14)	62.28 ± 13.02 60.24 ± 10.32	4.06 ± 2.34 3.74 ± 2.50
**Meng-Hsiang Chen et al., 2017** [79]	PD HC	29 (20) 26 (19)	61.51 ± 8.27 60.11 ± 7.77	NA
**Fuyong Chen et al., 2019** [80]	HC PD-CN PD-aMCI	20 (80%) 19 (78.9%) 17 (88.2%)	59.5 ± 6.2 61.3 ± 6.9 64.9 ± 5.9	_ 5.9 ± 3.4 7.6 ± 4.9
**Elisa Canu et al., 2015** [49]	PD-punding PD no-ICB HC	21 (18) 28 (19) 28 (19)	63.8 ± 8.8 63.6 ± 6.5 61.9 ± 8.3	9.4 ± 5.4 9.7 ± 5.4 _
**Chin-Song Lu et al., 2016** [81]	PD HC	126 (68) 91 (43)	62.0 ±7.6 59.8 ±7.2	8.2 ±6.1 _
**Wen Zhou et al., 2021** [82]	Responsive group Irresponsive group	15 (7) 11 (6)	67.13 ± 8.52 68.91 ± 7.08	3.93 ± 3.10 2.82 ± 2.75
**Mina Ansari et al., 2016** [83]	PD-RBD PD-non-RBD	23 (18) 31 (20)	59.43 ± 10.97 60.64 ± 8.65	7.95 ± 8.76 months 7.32 ± 8.19 months
**Suk Yun Kang et al., 2019** [84]	Without fatigue With fatigue	23 (10) 9 (8)	70.0 ± 8.4 63.6 ± 12.5	22.7 ± 28.5 months 27.6 ± 2.2 months
**Lauren Uhr et al., 2022** [85]	PD	31 (24)	64.5 (5.80)	8.48 (3.38)
**Jilu Princy Mole et al., 2016** [25]	PD HC	24 (20) 26 (17)	63.42 ± 10.82 64.88 ± 8.06	NA

**Table 2:** An overview of the literature regarding studies with significant microstructural changes of Cingulum in association with PD symptoms. (NA is a written abbreviation for not applicable.)

	Between-Group Findings				Symptomatology Correlations with DTI Metrics		Complementary Information of Participants	
**Study**	FA	MD	RD or AD increases	Additional imaging results	Applied tests	Significant associations	Drug exposure	Group matches
**Zonghong Li et al., 2020 [27]**	No significant differences	Non-depressed PD, depressed PD > HC in left uncinate fasciculus	RD: No significant differences AD: non-depressed PD, depressed PD > HC in right hippocampal part of cingulum and bilateral uncinate fasciculus	NA	HDRS, MMSE, UPDRS-III, H&Y	NA	NA	Age, sex, years of education or MMSE
**Martin Gorges et al., 2019 [29]**	PD < HC in cingulum	NA	NA	NA	H&Y, UPDRS-III, MMSE, PANDA, CERAD	Significant correlations between cognitive state-dependent regional FA changes and the sociodemographically corrected CERAD total score in cingulum	ON	
**Mattis Jalakas et al., 2019 [31]**	NA	NA	NA	NA	UPDRS-III, MMSE	Correlations between declining processing speed and discrepancies in the cingulum tract using the mean diffusivity, MD, parameter	ON	
**Christina Andica et al., 2019 [32]**	HC > PD in UF and cingulum hippocampus	HC < PD in UF	AD: HC < PD in UF RD: HC < PD in UF and cingulum hippocampus	NA	MDS-UPDRS, H&Y	NA	ON	
**Christina Andica et al., 2020 [33]**	HC > PD-woNCP in UF and right cingulum hippocampus HC > PD-wNCP in cingulum hippocampus and UF	HC < PD-woNCP in UF HC < PD-wNCPs in right cingulum hippocampus and UF	RD: HC < PD-woNCP in UF and cingulum hippocampus HC < PD-wNCP in UF and cingulum hippocampus AD: HC < PD-wNCP in UF	TOI: FA:PD-woNCP < HC PD-wNCP < HC MD: PD-woNCP > HC PD-wNCP > HC RD: PD-woNCP > HC PD-wNCP > HC	H&Y, UPDRS I, UPDRS-III, TOI	NA	ON	Age and sex-matched HCs
**Nan-Kuei Chen et al., 2018 [34]**	PD > HC in Cingulum (hippocampus)	NA	NA	NA	MMSE, UPDRS-III, H&Y	The FA values negatively correlated with the UPDRS-III scores across PD patients in Cingulum (hippocampus) and UF	NA	
**Haruka Takeshige-Amano et al., 2022** [35]	NA	PD-ICB > HC in uncinate fasciculus PD-nICB > PD-ICB in cingulum and UF	NA	NA	H&Y, UPDRS I, UPDRS-III	NA	ON	
**Jia-Yong Wu et al., 2017 [36]**	PDD < PDnD in UF and cingulum	NA	NA	NA	UPDRS-III, H&Y, MMSE, HDRS	FA values in the left cingulum and left superior longitudinal fasciculus of the PDD group were negatively correlated with HDRS scores, but no correlation was found with other disease characteristics including age, duration, UPDRS-III, H-Y scale, MMSE	NA	Age, age of onset, disease duration, sex
**Jingwen Chen et al., 2022 [37]**	LAR, LTD < HC No significant differences between RAR, RTD and HC in cingulum bundle	No significant differences	NA	NA	UPDRS-III, H&Y, MMSE	NA	ON	Age, disease duration, sex, Levodopa equivalent daily dos
**Gordon W. Duncan et al., 2015 [39]**	No significant difference	PD > HC in cingulum	NA	NA	MDS-UPDRS-III, H&Y, MMSE, MoCA	NA	ON	Age, gender, and education
**Abhishek Lenka et al., 2020 [40]**	PD-P < PD-NP in cingulum	No significant difference	No significant difference	NA	MoCA, HAMD, HAMA, H&Y, FAB, UPDRS-III, Corsi block-tapping test, RAVLT, CFT, TMT-B, Stroop effect	NA	ON	Age, sex, age of onset, disease duration
**Rémi Patriat et al., 2022 [41]**	NA	PD−RSWA < HC in cingulum	RD: PD−RSWA < HC in cingulum	NA	MDS-UPDRS-III, H&Y, MoCA	NA	ON	Sex, age, education and MoCA, age at diagnosis, years since diagnosis, H&Y AND total MDS UDRS III score
**Yuanjing Feng et al., 2020 [42]**	SWEDD < PD, HC PD > HC in cingulum bundle	NA	AD: SWEDD > PD, HC in cingulum bundle	NA	UPDRS-III, MoCA	NA	NA	
**Markus Nilsson et al., 2015 [43]**	PDD > HC in cingulum	NA	NA	NA	NA	NA	NA	
**Rachel P. Guimarães et al., 2018 [45]**	All PD < HC in cingulum	NA	RD: ALL PD > HC in cingulum SPD > HC, MPD, MoPD in cingulum	ROI: no FA difference between groups. AD and RD were higher in SPD when compared to HC, MPD and MoPD	UPDRS, UPDRS-PartIII, H&Y, SCOPA, SCOPA, NMSS	Positive association between SCOPA-COG scores and FA values, and a negative association with RD and UPDRS, UPDRS-III and NMSS, were positively associated with AD and RD values in cingulum	ON	Age and sex
**Chaoyang Jin et al., 2021 [46]**	PD FOG+ < HC PD FOG+ < PD FOG– in the cingulum	PD FOG– > HC in cingulum	NA	NA	UPDRS-III, H&Y, FOGQ, MMSE, MoCA, TUG, HDRS NA, HARS	NA	NA	
**Yunjun Yang et al., 2022 [47]**	dPD < ndPD in right cingulum (cingulate gyrus), left cingulum hippocampus	dPD > ndPD in right cingulum (cingulate gyrus)	NA	NA	H&Y, UPDRS- III, HAM-D, HAMA, MMSE, MoCA	NA	NA	
**Xiaojun Guan et al., 2018 [48]**	PD < HC only in the right UF	PD > HC in the left cingulum	RD: PD > HC AD: PD < HC	NA	UPDRS, H&Y, MMSE	In the right cingulate gyrus, significant correlation of increased MMS with disease duration	NA	Age and sex
**Elisa Canu et al., 2015 [49]**	PD-FoG < HC in cingulum	PD-FoG > HC in cingulum	NA	NA	FOGQ, UPDRS-III	NA	ON	Age, sex, education
**Gong-Jun Ji et al., 2019 [50]**	PD < HC in the left cingulum	NA	NA	NA	UPDRS-III, H&Y, MMSE, MoCA	No significant correlation was found between FA in the left cingulum and clinical measures	51 of PD patients OFF, 6 ON	Age, sex, and education-matched HCs
**Yiming Xiao et al., 2021 [52]**	PD > HC in cingulum Right dominant PD > Left dominant PD in cingulum	NA	NA	NA	H&Y, UPDRS, GDS, RBDSQ	NA	NA	
**Chih-Ying Lee et al., 2019 [53]**	PDSa < PDNsa in bilateral cingulum	PDSa > PDNsa in bilateral cingulum	RD: PDSa > PDNsa in bilateral cingulum	NA	UPDRS, H&Y, MMSE, S&E, ASMI	Significantly associations between ASMI and FA of the ROI in the left cingulum	ON	Age, gender, height, MMSE
**Hye Bin Yoo et al., 2015 [55]**	PD-ICD > PD-nonICD in right dorsal and posterior cingula	NA	NA	NA	MMSE, UDPRS, H&Y	NA	ON	Age, sex, MMSE score, GDS score, disease duration, total daily LED, UPDRS, HY stage
**Olaia Lucas-Jim´enez et al., 2015 [56]**	PD < HC in right ACT PD > HC in left PCT	NA	NA	NA	UDPRS, H&Y	In correlations between verbal memory and FA of the right PCT, FA correlated positively with correct rejections and negatively with false positives in HC group between brain activation in the left IOFC during the verbal learning memory fMRI task and FA of the right UF	ON	
**Xiang-Rong Li et al., 2018 [59]**	PD < HC in left unciform fasciculus, right cingulum	NA	NA	NA	H&Y, UPDRS, MMSE	UPDRS and motor score had no relationship with the FA of each white matter fasciculus	OFF	
**Virendra R. Mishra et al., 2019 [61]**	No significant differences	No significant differences	No significant differences	NA	MDS-UPDRS-III, MoCA, H&Y	TBSS:Negative correlation with disease duration and bilateral CGC DTI-TK: Positive correlation with disease duration and RD in left CGC	NA	Sex, age, years of education, and handedness and MoCA
**Thais Minett et al., 2018 [62]**	PDMCI < HC in cingula	PD-MCI and PD-N > HC in cingula	NA	NA	UPDRS-III, H&Y	NA	ON	Age, proportion of WML, duration of PD, levodopa equivalent dose
**Maria Chondrogiorgi et al., 2019 [63]**	PDD < PD-CTRL in cingulum (cingulate gyrus) and uncinate fasciculus	No significant differences	No significant differences	NA	H&Y, MMSE, HAM-D, PD-CRS	Lower total PD-CRS score was associated with FA decreases incingulum (cingulate gyrus), cingulum (hippocampus) and uncinate fasciculus	ON	Sex, years of education
**Yuko Koshimori et al., 2015** [64]	NA	PD > HC in cingulum and uncinate fasciculus	NA	NA	MoCA, UPDRS-III,	NA	ON	Age, sex, education, BDI, and handedness
**Ming-fang Jiang et al., 2015 [65]**	PD < HC in cingulum bundle	NA	NA	NA	H&Y, UPDRS-III, MoCA, ADL, HAMD	FA values in the white matter tracts showed no correlation with UPDRSIII scores	ON	Age and sex
**Sara Pietracupa et al., 2017** [66]	NA	HC < PD-FOG, PD-NFOG PD-FOG > PD-NFOG in UF HC, PD-FOG < PD-NFOG HC < PD-FOG in Cingulum angular bundle	RD: HC < PD-FOG, PD-NFOG PD-FOG < PD-NFOG in UF HC, PD-FOG < PD-NFOG HC < PD-FOG in Cingulum angular bundle AD: HC, PD-FOG < PD-NFOG HC > PD-FOG in UF	NA	H&Y, UPDRS-III, MMSE, FAB, HAM-D	DTI values in the uncinate fasciculus and cingulum (both cingulate gyrus and angular bundles) bilaterally significantly correlated with the cognitive scores, as assessed by the MMSE DTI values in the uncinate fasciculus and cingulum (angular bundle and cingulate gyrus) bilaterally significantly correlated with frontal abilities, as indicated by the FAB scores significant correlation between DTI values in the right uncinate fasciculus and the HAM-D scores	ON & OFF	Age and sex
**Laura Pelizzari et al., 2019 [68]**	No significant differences	RPD > HC in right cingulum LPD > HC RPD > LPD	No significant differences	NA	MDS-UPDRS-III, H&Y, MoCA	NA	ON	
**Tracy R. Melzer et al., 2015 [69]**	No significant differences	No significant differences	No significant differences	Time effects: FA showed widespread reduction in cingulum bundles, MD, and RD exhibited significant, yet more restricted increases	MDS-UPDRS-III	NA	ON	Age, education, sex
**Yulia Surova 2016 [70]**	PD > HC in Cingulum hippocampus	NA	NA	NA	UPDRS, H&Y, MMSE, AQT, ADAS-Cog	NA	NA	
**Morinobu Seki et al., 2019 [74]**	NA	PD, HC > MSA-P in adjacent cingulum	NA	NA	UPDRS-III, H&Y, MMSE	NA	NA	Sex, age, and disease duration
**Min Wang et al., 2016 [75]**	PD-FOG < HC in left cingulum	PD-FOG > HC in left cingulum	NA	NA	FOGQ, MMSE, UPDRS-III	NA	ON	Age, sex, education PD-FOG and PDnFOG: Disease duration, UPDRS-III, LEDD
**Ming-Ching Wen et al., 2018 [76]**	TD > HC in right cingulum TD > PIGD in right and left cingulum no significant between HC and PIGD	NA	RD: TD < HC in right cingulum, PIGD > TD in UF AD: PIGD > HC in UF	NA	UDPRS, H&Y, MOCA, GDS, Cardiovascular burden, Head motion	NA	OFF	Age, sex, education, handedness, dominant side, PD duration/H&Y scale, cardiovascular burden, head motion
**Jingqiang Wang et al., 2020 [77]**	Significant Difference in UF	NA	AD: Significant Difference in cingulum bundle		MoCA, UPDRS-III, GDS	Cingulum bundle have correlation with GDS	NA	
**Yang Zhang et al., 2017 [78]**	apathy group < Non-apathy in left cingulum	NA	NA	NA	H&Y, UPDRS-III, MMSE, BDI-II	FA and LARS scores were negatively correlated in left cingulum	ON	Age, sex, disease duration, LEDD, UPDRS-III, MMSE
**Meng-Hsiang Chen et al., 2017 [79]**	PD < HC in left cingulum	NA	NA	NA	H&Y, UPDRS-III, S&E	MD values in the left cingulum were positively correlated with baroreflex sensitivity and negatively correlated with serumnuclearDNA simultaneously AD values in the left cingulum were positively correlated with the serum nuclear DNA level RD values in the left cingulum were simultaneously positively correlated with baroreflex sensitivity and negatively correlated with serum nuclear DNA	NA	Age and sex
**Fuyong Chen et al., 2019 [80]**	PD-CN > PD-aMCI in cingulum (cingulate gyrus) in the bilateral hemispheres	NA	NA	NA	H&Y, UPDRS-III, MMSE, RBANS	NA	ON	
**Chin-Song Lu et al., 2016 [81]**	NA	NA	NA	NA	H&Y, UPDRS-III, MMSE, ADL	A statistically significant association between ADL and maximum MD/RD in the ipsilateral posterior cingulum	OFF	
**Wen Zhou et al., 2021 [82]**	Irresponsive group < Responsive group in bilateral cingulum	NA	NA	NA	H&Y, UPDRS-III, MMSE	NA	ON	Parkinson’s disease duration, age, sex
**Mina Ansari et al., 2016 [83]**	PD-RBD < PD-non-RBD in cingulum	NA	NA	NA	RBD, MOCA, GDS, UPDRS-III, H&Y, ESS, LNS	NA	NA	Age, sex
**Suk Yun Kang et al., 2019 [84]**	PD < PD with fatigue in right cingulum, UF	PD > PD with fatigue in right cingulum	RD: PD > PD with fatigue in UF, cingulum	NA	K-MMSE, MoCA, BDI, UPDRS, H&Y, FSS	NA	NA	
**Lauren Uhr et al., 2022 [85]**	NA	NA	NA	NA	UPDRS	Positive ROI-based correlations of FA and depressive symptoms in left cingulum (hippocampus)	ON	

**Table 3:** An overview of the literature regarding studies with significant microstructural changes in the uncinate fasciculus in association with PD symptoms. (NA is a written abbreviation for not applicable.)

	Between-Group Findings				Symptomatology Correlations with DTI Metrics		Complementary Information of Participants	
**Study**	FA	MD	RD or AD increases	Additional imaging results	Applied tests	Significant associations	Drug exposure	Group matches
**Boyu Chen et al., 2015** [26]	PD-Cu > PDD inbilateral uncinate fasciculus	PDD > PD-Cu in bilateral uncinate fasciculusHC > PDD	NA	NA	UPDRS-III, H&Y, MoCA, MMSE	MD value is negatively correlated with MoCa scores in UF	NA	
**Zonghong Li et al., 2020** [27]	No significant differences	Non-depressedPD, depressed PD > HC in left uncinate fasciculus	RD: No significant differencesAD: non-depressed PD, depressed PD > HC in right hippocampal part of cingulum and bilateral uncinate fasciculus	NA	HDRS, MMSE, UPDRS-III, H&Y	NA	NA	Age, sex, years of education orMMSE
**Florian Holtbernd et al., 2019** [28]	PD, RBD > HC in left uncinate fasciculus	No significant differencebetween RBD, PD, and HC	No significant differencebetween RBD, PD, and HC	NA	MDS UPDRS-III, MoCA, H&Y	NA	ON	Age-matched HCs
**Junyan Sun et al., 2021** [30]	NA	PD > HC inbilateral uncinate fasciculus	NA	NA	H&Y, UPDRS-III	NA	NA	Age, sex, education-matched HCs
**Christina Andica et al., 2019** [32]	HC > PD in UF and cingulum hippocampus	HC < PD in UF	AD: HC < PD in UFRD: HC < PD in UF and cingulum hippocampus	NA	MDS-UPDRS, H&Y	NA	ON	
**Christina Andica et al., 2020** [33]	HC > PD-woNCP in UF and right cingulum hippocampusHC > PD-wNCPin cingulum hippocampus and UF	HC < PD-woNCP in UFHC < PD-wNCPs inright cingulum hippocampus and UF	RD: HC < PD-woNCP in UF and cingulum hippocampusHC < PD-wNCP in UF and cingulum hippocampusAD: HC < PD-wNCP in UF	TOI:FA:PD-woNCP < HCPD-wNCP < HCMD: PD-woNCP > HCPD-wNCP > HCRD: PD-woNCP > HCPD-wNCP > HC	H&Y, UPDRS I, UPDRS-III, TOI	NA	ON	Ageand sex-matched HCs
**Nan-Kuei Chen et al., 2018** [34]	PD > HCin Cingulum (hippocampus)	NA	NA	NA	MMSE, UPDRS-III, H&Y	The FA values negativelycorrelated with the UPDRS-III scores across PD patients in Cingulum (hippocampus) and UF	NA	
**Haruka Takeshige-Amano et al., 2022** [35]	NA	PD-ICB > HC in uncinate fasciculusPD-nICB > PD-ICB in cingulum and UF	NA	NA	H&Y, UPDRS I, UPDRS-III	NA	ON	
**Jia-Yong Wu et al., 2017** [36]	PDD < PDnD in UF and cingulum	NA	NA	NA	UPDRS-III, H&Y, MMSE, HDRS	FA values in the left cingulumand left superior longitudinal fasciculusof the PDD group were negatively correlatedwith HDRS scores, but no correlation was found with otherdisease characteristics including age, duration, UPDRS-III, H-Y scale, MMSE	NA	Age, age of onset, disease duration, sex
**Sonia Di Tella et al.,2020** [38]	No significant differences	PD > HC in left UF	NA	NA	H&Y, UPDRS-III	FA of the left UF was positively correlated with the accuracy in theglobal word production (N + V), N production, Vproduction and semantic fluencyFA of the right UF was positively correlated with the global word production and N productionno significant correlations were observedbetween FA and MD and the three measures of production task (N, V and N + V production)	ON	Age, sex, disease duration and years of education
**Lubin Gou et al., 2018** [44]	All PD < HC in left uncinate fasciculus	NA	NA	NA	MoCA, MDS-UPDRS-III, H&Y	NA	OFF	Sex, age, MoCA, and educationyears
**Xiaojun Guan et al., 2018** [48]	PD < HC only inthe right UF	PD > HC in theleft cingulum	RD: PD > HCAD: PD < HC	NA	UPDRS, H&Y, MMSE	In the right cingulate gyrus, significant correlation of increasedMMS with disease duration	NA	Age and sex
**Tao Guo et al., 2020** [51]	No significantdifferences in the FA	S-depression > HC inuncinate fasciculusNo difference in the MD among the other pairs of comparisons	NA	NA	GCO, PDQ-39, UPDRS, H&Y, MMSE	NA	ON	Age, sex, education
**F Imperiale et al., 2017** [54]	PD-ICB < PD no-ICB, HCin left uncinate fasciculus	PD-ICB > PD no-ICB, HCin right uncinate fasciculus	NA	NA	QUIP, H&Y, UPDRS-III, HDRS	NA	ON	All matched in:Age, sexEducationPatients matchedin age at PDonset diseasedurationSide of onsetH&Y scoresUPDRS-IIIcognitive status
**Olaia Lucas-Jim´enez et al., 2015** [56]	PD < HC in right ACTPD > HC in left PCT	NA	NA	NA	UDPRS, H&Y	In correlations between verbal memory and FA of the right PCT, FA correlated positivelywith correct rejections andnegatively with false positives in HC groupbetween brain activation in the left IOFC during the verbal learning memory fMRI task and FA of theright UF	ON	
**Takahiro Koinumaa 2021** [57]	HC < PARK2 inuncinate fasciculus	RD: HC < PARK2 inuncinate fasciculus	NA	NA	UDPRS III, H&Y	In PRKN AD values were negatively correlatedwith the serum levels of 9-hydroxystearate, while the MD and RD values were positivelycorrelated with these levels in UF	ON	Sex, age, cerebrovascular risk factors
**Charalampos Georgiopoulos et al., 2017** [58]	NA	NA	AD: PD < HC in left uncinate fasciculus	NA	UPDRS-III, H&Y, MMSE	NA	ON	Age, sex
**Kazufumi Kikuchi et al., 2017** [60]	PD-MIBGL < PD-MIBGH in left uncinate fasciculus	No significantdifferences	NA	NA	MDS, H&Y, MMSE	NA	ON	Age, sex, diseaseduration, MMSE, H&Y stage
**Maria Chondrogiorgi et al., 2019** [63]	PDD < PD-CTRL in cingulum (cingulategyrus) and uncinate fasciculus	No significantdifferences	No significantdifferences	NA	H&Y, MMSE, HAM-D, PD-CRS	Lower total PD-CRS score was associated with FA decreases incingulum (cingulate gyrus), cingulum (hippocampus) and uncinate fasciculus	ON	Sex, years of education
**Yuko Koshimori et al., 2015** [64]	NA	PD > HC in cingulum anduncinate fasciculus	NA	NA	MoCA, UPDRS-III,	NA	ON	Age, sex, education, BDI and handedness
**Sara Pietracupa et al., 2017** [66]	NA	HC < PD-FOG, PD-NFOGPD-FOG > PD-NFOGin UFHC, PD-FOG < PD-NFOGHC < PD-FOG in Cingulum angular bundle	RD: HC < PD-FOG, PD-NFOGPD-FOG < PD-NFOGin UFHC, PD-FOG < PD-NFOGHC < PD-FOG in Cingulum angular bundleAD: HC, PD-FOG < PD-NFOGHC > PD-FOG in UF	NA	H&Y, UPDRS-III, MMSE, FAB, HAM-D	DTI values in the uncinate fasciculus and cingulum(both cingulate gyrus and angular bundles) bilaterallysignificantly correlated with the cognitive scores, as assessedby the MMSEDTI values in the uncinate fasciculus and cingulum(angular bundle and cingulate gyrus)bilaterally significantly correlated with frontal abilities, asindicated by the FAB scoressignificant correlation between DTI values in the right uncinate fasciculus and the HAM-D scores	ON and OFF	Age and sex
**A. Inguanzo et al., 2020** [67]	PD1 < HC inuncinate fasciculus	NA	NA	NA	UPDRS-III, H&Y, MMSE	NA	ON	Sex andyears of education
**Takashi Ogawa 2021** [71]	PD-nLID < HCPD-nLID < PD-LID in uf	NA	NA	NA	MDS-UPDRS, H&Y	NA	ON	Age, sex
**Jinqiu Yu et al., 2022** [72]	G/G > G/A in UF	NA	RD: G/G < G/A in UF	NA	MMSE, MoCA, H&Y, UPDRS-III	NA	ON	
**Qin Shen et al., 2021** [73]	sdPD < ndPD in UFsdPD < mdPD in UFNo significant difference between ndPD and mdPD	NA	RD: sdPD > ndPD in UF	NA	H&Y, MMSE, CDR, UPDRS-III, BDI	Nosignificant correlation was found between BDI scores andFA values in other tracts.	NA	Age, sex, education, CDR, MMSE, PD duration, H&Y scales, and UPDRS-III scores
**Ming-Ching Wen et al., 2018** [76]	TD > HC in right cingulumTD > PIGD in right and left cingulumno significant between HC and PIGD	NA	RD: TD < HC in right cingulum, PIGD > TD in UFAD: PIGD > HC in UF	NA	UDPRS, H&Y, MOCA, GDS, Cardiovascular burden, Head motion	NA	OFF	Age, sex, education, handedness, dominant side, PDduration/H&Y scale, cardiovascular burden, head motion
**Jingqiang Wang et al., 2020** [77]	SignificantDifference in UF	NA	AD: SignificantDifference in cingulum bundle		MoCA, UPDRS-III, GDS	Cingulum bundle have correlation with GDS	NA	
**Elisa Canu et al., 2015** [49]	NA	PD-punding > HC in right uncinate fasciculus	NA	NA	H&Y, UPDRS-III, MMSE, HAMA, HDRS	NA	ON	Age, sex, and education
**Suk Yun Kang et al., 2019** [84]	PD < PD with fatigue in right cingulum, UF	PD > PD with fatigue in right cingulum	RD: PD > PD with fatigue in UF, cingulum	NA	K-MMSE, MoCA, BDI, UPDRS, H&Y, FSS	NA	NA	
**Jilu Princy Mole et al., 2016** [25]	PD < HC in UF	PD > HC in left UF	RD: PD > HC in right UF	NA	UDPRS, H&Y, MOCA	NA	ON	

## Data Availability

Not applicable.
